# A Massive Posterior Neck Mass: Lipoma or Something More Sinister?

**DOI:** 10.1155/2013/205936

**Published:** 2013-10-08

**Authors:** Matthew F. Ryan, Brandon Allen

**Affiliations:** Department of Emergency Medicine, University of Florida, 1329 SW 16th Street, P.O. Box 1000186, Gainesville, FL 32610-0186, USA

## Abstract

Lipomas are slow-growing benign soft-tissue tumors which are typically asymptomatic and occur in approximately 1% of the population. A lipoma is considered to be of excessive size when it is greater than 10 cm in length (in any dimension) or weighs over 1000 g (Kransdorf (1995)). We describe a case of a man presenting with a giant posterior neck mass which greatly reduced the sagittal range of cervical spine. A discussion of the pathophysiology of lipomas and a literature review regarding giant lipomas versus malignancy follows.

## 1. Introduction

Lipomas are slow-growing benign soft-tissue tumors which are typically asymptomatic and occur in approximately 1% of the population [1]. Lipomas are often small and solitary lesions and can grow in any area of the body where adipose tissue is present. Whereas the majority of lipomas grow on the extremities and trunk, only 13% are reported to form on the neck and the head [2]. The tissue in a lipoma is mature typically adipose which often forms in a septated lobules encased in fibrous connective tissue. A lipoma is considered to be of excessive size when it is greater than 10 cm in length (in any dimension) or weighs over 1000 g [3]. We describe a case of a man presenting with a giant posterior neck mass which greatly reduced the sagittal range of cervical spine. A discussion of the pathophysiology of lipomas and a review of the literature regarding giant lipomas versus malignancy follows.

## 2. Case

A 32-year-old man presents to the emergency department with the chief complaint of pain and discomfort caused by a large posterior neck mass. The patient states that he noticed the mass for a few months and now it has grown to the point where he can no longer fully extend his neck. He has constant midline neck pain, headaches, and parasthesias in his fingers bilaterally which began approximately one week prior to presentation. The size of the lipoma inhibited the patient's ability to look directly up without turning his head sideward. The patient's pain, decreased range of motion, and parasthesias have gradually worsened to the point where his symptoms are aggravated by any movement of his head or neck. He denies trauma or recent injuries and insists that he had not noticed any other symptoms until he “slept funny” about two months ago. The patient also denies fevers or constitutional symptoms and reports no other neurological deficits other than parasthesias. 

Physical exam reveals a well-developed (BMI = 27), well-appearing man who looks his stated age. His heart rate, blood pressure, and respiratory rate are within normal limits and he was afebrile. Examination of the head and neck reveals no lymphadenopathy, a clear oropharynx, and no cranial nerve deficits. A large posterior neck mass was noted spanning from the occiput to the level of T2 obscuring the vertebra prominens. The mass was doughy, mobile, and compressible (which elicited pain) and lacked evidence of an infectious process demonstrating no redness, induration, or fluctuance. The patient reports posterior neck tenderness upon palpation with decreased range of motion in the neck—especially to extension. There is no tracheal deviation, no erythema is present, and meningismus was absent. The patient had 5 of 5 strength in his arms and hands but decreased sensation in his hands bilaterally. The remainder of the physical exam was normal. 

**Figure 1:**
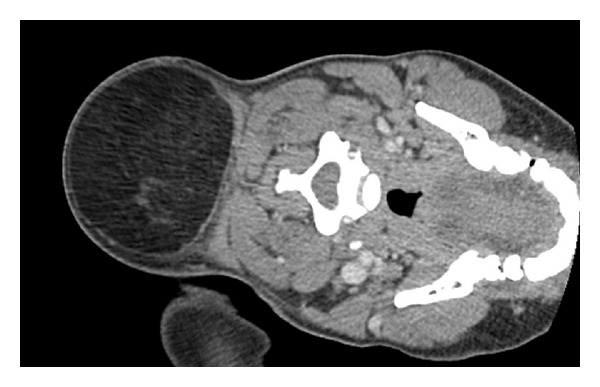
CT image showing a slice of a large posterior cervical lipoma which spanned from the patient's occiput through the length of the patient's cervical spine.

The mass appeared to be a giant lipoma although the differential diagnosis also included liposarcoma, cyst, abscess, or other soft-tissue lesions. Because of the concern for an invading mass into the paraspinal region of the cervical spine, especially considering the patient's level of pain and neurological deficits in his upper extremities, a noncontrast computed tomography scan of the neck was ordered for further evaluation of the mass. The imaging study (Figure 1 showing slice 36 of 79) revealed a well-circumscribed encapsulated fat-containing lesion of the posterior neck measuring 8 cm × 9 cm × 10 cm (total volume ca. 720 cm^3^). The mass itself was clearly superficial to the cervical spine, spinal canal, and the paravertebral musculature and showed no calcification, internal hemorrhage, or evidence for surrounding inflammation. A referral was made to the plastic surgery clinic for evaluation and possible surgical debulking of a symptomatic lipoma. 

## 3. Discussion

The patient, by definition, had a giant lipoma of the posterior neck. In the recent medical literature, there are only two previous case reports of a lipoma versus mass of the posterior neck [1, 2]. Ali et al. reported the dangers of a giant lipoma in the setting of airway management [4]. A mass of this size will inhibit neck extension and may obstruct the view of the larynx. Our patient described previously had no indication for airway involvement, but the patient with a giant lipoma of the posterior neck would need preparation for a failed airway and the most experienced intubator to perform the intubation. The second paper discussed the sometimes subtle differences between a lipoma and liposarcoma. Jones et al. cautioned that there are specific criteria that would make a liposarcoma more likely including evidence of calcifications, size greater than 10 cm, and irregularly, thickened septae [5]. Fortunately, our patient displayed none of these findings on noncontrast computed tomography. However, he did possess a significant decrease in neck extension and was unable to elevate his head more than ca. 30° above the horizon. This led to him being symptomatic in regards to his range of motion and thus quality of life. In closing, the differential diagnosis of a large posterior neck mass is broad with potentially devastating sequelae. The diagnosis of giant lipoma is in fact a diagnosis of exclusion. This case underscores the importance of advanced imaging to differentiate lipoma from liposarcoma and other potentially life-threatening complications. On a recent review of the patient's chart, he has not been seen in the plastic surgery clinic, and excision/debulking has not occurred.
